# An Interactive Tool for Outdoor Computer Controlled Cultivation of Microalgae in a Tubular Photobioreactor System

**DOI:** 10.3390/s140304466

**Published:** 2014-03-06

**Authors:** Raquel Dormido, José Sánchez, Natividad Duro, Sebastián Dormido-Canto, María Guinaldo, Sebastián Dormido

**Affiliations:** Department of Computer Sciences and Automatic Control, UNED, C/Juan del Rosal, 16, 28040 Madrid, Spain; E-Mails: jsanchez@dia.uned.es (J.S.); nduro@dia.uned.es (N.D.); sebas@dia.uned.es (S.D.-C.); mguinaldo@dia.uned.es (M.G.); sdormido@dia.uned.es (S.D.)

**Keywords:** photobioreactors, biocontrol, biotechnology, laboratory education, simulators, interactive programs, modeling, Java

## Abstract

This paper describes an interactive virtual laboratory for experimenting with an outdoor tubular photobioreactor (henceforth PBR for short). This virtual laboratory it makes possible to: (a) accurately reproduce the structure of a real plant (the PBR designed and built by the Department of Chemical Engineering of the University of Almería, Spain); (b) simulate a generic tubular PBR by changing the PBR geometry; (c) simulate the effects of changing different operating parameters such as the conditions of the culture (pH, biomass concentration, dissolved O_2_, inyected CO_2_, *etc.*); (d) simulate the PBR in its environmental context; it is possible to change the geographic location of the system or the solar irradiation profile; (e) apply different control strategies to adjust different variables such as the CO_2_ injection, culture circulation rate or culture temperature in order to maximize the biomass production; (f) simulate the harvesting. In this way, users can learn in an intuitive way how productivity is affected by any change in the design. It facilitates the learning of how to manipulate essential variables for microalgae growth to design an optimal PBR. The simulator has been developed with Easy Java Simulations, a freeware open-source tool developed in Java, specifically designed for the creation of interactive dynamic simulations.

## 1. Introduction

Microalgae are of increasing interest for research and industry. Microalgae culture for energy production has emerged as an interesting alternative to fossil fuels and biofuels [1,2]. Its great potential to produce a wide range of important biochemical components for food or medicine is also well known [3]. Even though in the last years microalgae biomass has generated overwhelming interest, cultivation of microalgae is not an easy task. Difficulties arise from, e.g., the need to avoid contamination, the energy supply and cultivation conditions. For example, high light intensities can lead to photo- inhibition, but low intensities probably limit the photosynthesis activity and other related cellular processes.

Present only a few hundred tons of products are produced in closed photobioreactors (PBR for short). One of the most important hindrances to a sustainable commercial use of algae cultures is the lack of suitable PBRs to guarantee optimal microalgae growth conditions.

The design and development of a PBR for maximum production of microalgae involves many questions: detailed knowledge of light distribution, mass transfer, scalability, *etc.* Apart from maximum production, other factors such as design, cost effectiveness, low maintenance costs or energy requirements need to be optimized. To date, no PBR fulfills all these requirements.

On the other hand, process sensing is an essential and integral component of PBR technology. Industrial PBR systems demand accurate tracking of multiple culture parameters to correctly reflect the process dynamics, and from which appropriate actions can be implemented to enhance process performance. In addition to the standard sensors for temperature, dissolved oxygen, and pH, many sophisticated electronic sensors for measuring a variety of culture and process parameters (such as viability, metabolic activities, biomass and nutrient concentrations) are available. However, most of these sensors are limited by high cost, poor long-term stability, or both. Furthermore, installation of the required manifold of electronic hardware sensors would make the PBR system prohibitively expensive not only in terms of design and construction, but also maintenance.

These considerations suggest the necessity of advanced simulation tools in order to let researchers study new methods to improve the productivity. These tools would help investigate in depth the design and control of optimal PBRs to enable production under controlled and commercially competitive conditions.

The field of biotechnology is characterized by rapid changes in terms of novelty and by highly sophisticated processes (for instance, PBRs) that require careful design, operation and control in order to be run under safe and optimal conditions. Therefore facing a highly competitive global economic environment operational excellence is the key to exploiting these opportunities. This has to be added to the inherent complexity of the biological systems. To cope with all these issues innovative, reliable, smart, and cost-effective manufacturing processes are demanded, but, to meet these requirements involves the education of new engineers and vocational training students with tools that take advantage of the application of novel information and communication technologies to education in biotechnology and bioprocesses.

The use of computer simulations in the control process field to build models or to model real-world phenomena in order to help students gain insights into the behaviour of complex systems is of growing importance. Prominent advantages of virtual laboratories are not new [4–6]. Interacting with a simulation in virtual laboratories enable learners to gain better understanding of real systems, processes or phenomena through exploration, testing of hypotheses, and discovering explanations for processes.

In this context, the development of PBRs simulators tailored to the student profile is necessary. To date and to the authors' knowledge, no virtual laboratory for a PBR system has been developed. For vocational training students or plant operators, the development of virtual laboratories to master the operation of a PBR more from a qualitative view that from a quantitative and engineering perspective is justified. For the control and chemical engineering student, PBR simulators are fundamental to appreciate the complexity of these multivariable biological processes.

The advantages of a PBR interactive simulator, such as the one described in this paper, are clear, either for students or from an industrial point of view. It provides opportunities for users to modify their mental models, by comparing the outputs of the model with their expectations, and also it engages or motivates students to explore different effects which will lead to understanding. From an industrial point of view, the necessity of more advanced simulations PBR environments is compulsory. Many design factors must be optimized and balanced to implement an algae growing system in a large commercial facility.

The paper is organized as follows: Section 2 describes the PBR system. Section 3 presents the real system overview. In Section 4 some environmental issues are shown. Section 5 presents the PBR system developed in Easy Java Simulations (EJS for short). The paper ends with some concluding remarks and considerations about further works.

## 2. The Photobioreactor System

### 2.1. Photobioreactor Design

Mass cultivation of microalgae requires an appropriate culture system. There are different technical solutions for such cultivation [7,8]. Basically they can be classified in open PBR (known as raceways), which are open to the air, and closed PBR.

#### 2.1.1. Open Systems

The benefits of open systems lie in the ease of construction. They are also less expensive to build and maintain than closed systems. An open system can be in the form of a lake, pond, or some large open vessel that can hold water. Major limitations of this design include: (a) its susceptibility to evaporation; (b) the contamination by invasive species, which could take over the pond requiring draining and/or treatment; (c) the requirements for large areas of land; (d) temperature fluctuations that can affect algal growth; (e) in addition, once microalgae flourish, it is also difficult to get the maximum amount of light to all the microalgae since the pools are somewhat deep and efficient circulation is problematic.

#### 2.1.2. Closed Systems

Closed PBRs create an enclosed growing environment for algae cultivation where light, air, and nutrients are supplied at regulated levels to ensure optimized growth. Some benefits of these closed systems are: (a) microalgae cultures can grow free of potential contaminants such as microorganisms; (b) they provide higher production rates than open systems; (c) they are easier to manipulate and control, allowing the optimization of the essential variables and providing high growth rates; (d) they have less evaporation than open systems; (e) interior lighting can be adjusted for proper exposure levels. Problems with closed systems involve mainly the fact they are more expensive to set up and the facilities require greater amounts of maintenance.

**Figure 1.**
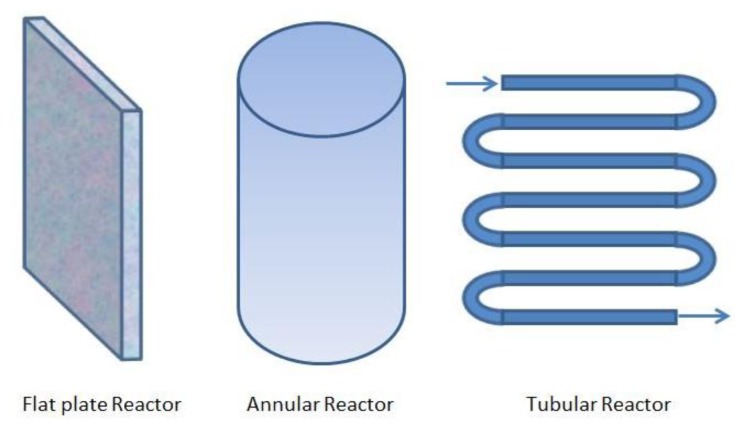
Commonly employed reactor designs.

An important factor of PBRs to maximize the growth conditions is their design, that is, the geometry employed to get an efficient distribution of light. The most common closed PBR geometries are the flat plate, the annular, and the tubular ones (see Figure 1). A complete description of these categories is given in [9].

Flat plate PBRs have received much attention due to their large illumination surface area. Generally, these PBRs are made of transparent materials for maximum utilization of solar light energy. Accumulation of dissolved oxygen concentrations in flat rates PBRs is relatively low compared to tubular PBRs. It has been reported that with flat plate PBRs high photosynthetic efficiencies can be achieved [10–12].

Annular reactors are typically translucent large diameter containers filled with algae suspended in a liquid medium, in which gases are bubbled from the bottom of the container. Since no precisely defined flow lines are reproducibly formed, it can be difficult to control the mixing properties of the system which can lead to low mass transfer coefficients, poor photomodulation, and low productivity. Moreover, to work with sufficient volume, the large diameter leads to a considerable dark fraction in the middle of the cylinder. This part does not contribute to productivity or may even have detrimental effects on growth.

The tubular PBR is one of the most suitable types for outdoor mass cultures. Most of them are usually constructed with either glass or plastic tubes. They consist of straight, coiled or looped transparent tubing arranged in various ways for maximizing sunlight capture. Properly designed tubular PBRs completely isolate the culture from potentially contaminating external environments, allowing extended duration algal cultures. Mixing of the cultures in tubular PBRs is usually done either with air-pump or airlift systems. This design is very suitable for outdoor mass cultures of algae since they have a large illumination surface area. On the other hand, one of the major limitations of tubular PBRs is their poor mass transfer. It should be noted that mass transfer (oxygen build-up) becomes a problem when tubular PBRs are scaled up. For instance, some studies have shown that very high dissolved oxygen levels are easily reached in tubular PBRs.

Comparing the three ways of engineering closed PBRs, the tubular ones facilitate better control of many culture environment parameters such as the carbon dioxide supply, the water supply, the optimal temperature, the efficient exposure to sunlight, the culture density, the pH level, the gas supply rate, or the mixing regime.

### 2.2. Production Cycle in a Closed PBR Tubular System

**Figure 2.**
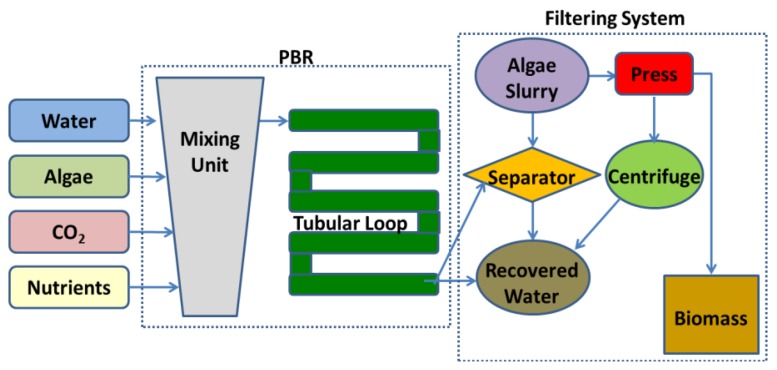
Schematic of a tubular PBR for outdoor culture.

Even though not all PBRs look alike, they basically work in the same way. A working schematic of a generic tubular PBR system for outdoor microalgae culture is shown in Figure 2.

An outdoor microalgae culture only needs nutrients, sunlight, and carbon dioxide to grow. From a mixing unit, the culture flow usually progresses to a pump which moderates the flow into the tube. Built into the pump is the CO_2_ inlet valve. The tubular loop itself, which acts as a solar receiver, is used to promote the biological growth by controlling the environmental parameters. Different sensors are usually available in the tubular loop such as oxygen sensors to determine how much oxygen has built up in the plant, or pH or temperature sensors. Once microalgae have completed the flow through the tubular loop, they pass back to the mixing unit. Efficient mixing should be provided in this unit in order to produce a uniform dispersion of microalgae within the culture medium, thus eliminating light gradients or for distributing the nutrient concentration. Likewise air is bubbled through the bottom of the mixing unit to provide good overall mixing, sufficient supply of CO_2_, and efficient removal of O_2_. At this stage, an optical sensor determines the harvesting rate. When the microalgae are ready for harvesting, they pass through the connected filtering system. This filter collects the microalgae ready for processing, while the remaining microalgae pass back to the mixing unit. After harvesting water with nutrients can be added to the mixing unit.

Another system incorporated into many PBRs is a built-in cleaning system that internally cleans the tubes without stopping the production. This is an agitation system which prevents the microalgae from sticking to the walls of the vessel and diminishing the amount of available light.

## 3. Real System Overview

### 3.1. Description of the plant

**Figure 3.**
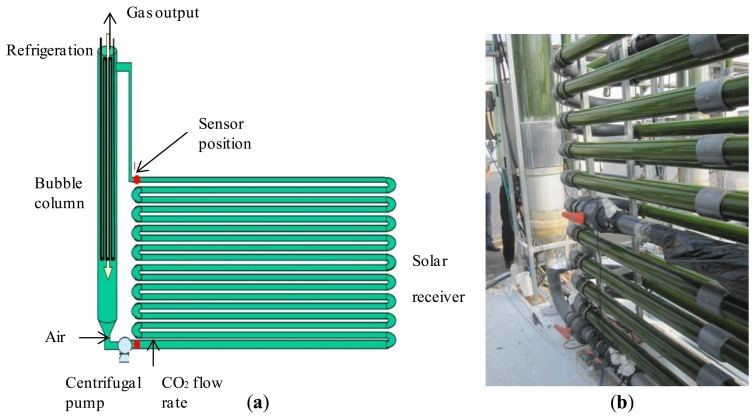
(**a**) Schema of the process. (**b**) Partial view of the mixing unit and the tubular loop of the PBR located in Almería (Spain).

The virtual laboratory described in this paper is based in the microalgae production facility located in “Experimental Station of Las Palmerillas”, property of the CAJAMAR Foundation (Almería, Spain; see Figure 3a,b).

The facility consists of 10 tubular fence-type PBRs built as described in [13–15]. Each PBR is made of a 400 m-long tube of 0.09 m diameter, with a bubble column 3.5 m high and 0.4 m in diameter for degassing and heat exchange. The tube diameter was optimized to maximize the culture volume per reactor, but minimizing yield losses caused by an excessive light path for photosynthesis. The tubes are optimally arranged to maximize the interception of solar radiation.

The tubular PBR model consists of two parts, which are a solar receiver (a continuous tubular loop) and a mixing unit (a bubble column). The culture is continuously recirculating from one to the other part using airlift and mechanical pumps. As the mass transfer and fluid-dynamics phenomena are different in each part, they are modeled separately.

In the bubble column both liquid and gas phases occur. On the one hand, the bubble (airlift) column circulates the culture through the solar receiver, where most of the photosynthesis happens. On the other hand, the oxygen produced by photosynthesis accumulates in the broth until the culture returns to the airlift zone where the accumulated oxygen is stripped by air (oxygen desorption) in order to prevent gas bubbles from recirculating into the solar receiver. In the external loop, the liquid circulates by a centrifugal pump where pure CO_2_ is supplied on demand for pH control purposes. Perfect mixing is considered in the liquid and gas phases. Cooling water pumped through a heat exchanger coil in the mixing unit is used for temperature control.

The complete system, as well as the data capture system and the control software which actually control and monitor all the facility activity, were designed and built by the Department of Chemical Engineering at the University of Almería (Almería, Spain).

### 3.2. Measurement and Performance Instrumentation

In the PBR system process sensing is an essential issue. In industrial PBRs it is desirable to reflect in a correct way the process dynamics in order to implement correct control actions to enhance the process performance. To this end many sensors and actuators are incorporated into the system for measuring a variety of culture and process variables (such as biomass, nutrient, concentrations, *etc.*).

The industrial PBR system on which this virtual laboratory has been inspired is endowed with different measurement and performance units. The sensors can be grouped as follows:

1. *Temperature sensors*: To measure the water temperature at the inlet and outlet of the heat exchanger, the average temperature of inlet water, the temperature of the mixing unit, the temperature of the culture, the ambient temperature, and the solar radiation.
2. *Flow sensors*: Different digital flowmeters are used to measure the CO_2_ flow, the air flow supplied to the bubble column and the heat exchanger inlet and outlet water flow.
3. *pH sensors*: Three different sensors placed at the entry of the column, in the central area of tubes and just before the point of the CO_2_ injection.
4. *Dissolved oxygen sensors* at the upper and bottom part of the column.
5. *Turbidity of the concentration of biomass sensor*: Located between the pump and the CO_2_ injection.

Although the PBR has all these sensors, the model implemented to control the system only uses a few of them. These are the three pH sensors, three temperature sensors situated in the external loop (one on top, one at the bottom, and the last at the halfway point of the loop), and the two dissolved oxygen sensors. It must be noted that this control system design (number of sensors, local controllers, *etc.*) is specific for this particular PBR design, but it can be easily extended for a different system.

In addition, there are a series of specific measures required for the operation. For example: the ambient temperature, the temperature at the inlet and outlet of the exchanger, the medium temperature water, the turbidity, the molar flow of CO_2_, the solar radiation, the air flow, the flow rate of cooling water, the medium flow, the flow of CO_2_, and the frequency of the CO_2_ injection pump. For all the above mentioned measures, sensors are used.

In order to use all these sensors, the PBR has a series of actuators such as a proportional valve (1–5 volts) flow CO_2_ input, a proportional valve for water in the heat exchanger, a proportional flow valve medium (nutrient), a frequency inverter in the internal fluid of the reactor recirculation, a proportional valve for air supply into the bubbling column.

From the actuators' point of view is important to note that the pump drive of the culture is driven by a frequency inverter which achieves the desired speed (set to 0.9 m/s). In addition, the entry of CO_2_ is controlled by a DC solenoid valve located just after the pump, so that the inflow is regulated manually by means of a rotameter (this flow has a value of 3 or 5 L/min). Moreover, the air in the column is controlled by a continuous valve, whose typical value is set at 80 L/min. Finally, the input to the heat exchanger flow is regulated by a continuous valve and this in turn is connected to two proportional valves, one for hot water and one for cold water, while the flow of medium is introduced into the reactor by means of a continuous valve located at the top of the column. Both performance measurement signals can be handled in real time in a way that is easy and simple, using a data acquisition module implemented by the Department of Chemical Engineering of Almería University.

### 3.3. The Mathematical Growth Model

Any model for a microalgae production system must consider the relationship between light availability and photosynthesis rate, the mixing and the gas–liquid mass transfer inside the system [15]. Bearing in mind these principles, a general growth model for photosynthetic microorganisms in PBRs can be developed, irrespective of the PBR design. The model of the PBR in this paper is based on mass balances, transport phenomena and equations simulating the biological phenomena taking place inside the culture.

The modeling of the tubular PBR can be divided into two main parts. The first part is the column, which can be approximated to a perfectly mixed reactor and where the composition of the output current is equal to the composition at any point within the reactor. The second part of the model is the external loop regarded as a flow reactor piston, wherein the composition of each differential volume varies relative to the length of the reactor. Perfect mixing is assumed in each differential element. The number of differential elements in the virtual laboratory is a parameter that can be interactively changed when the user wants to change the precision of the results.

The model includes mass balances in both the liquid and gas phase. For the liquid phase, changes in dissolved oxygen concentration are related to the gas-liquid mass transfer rate and the photosynthesis rate. Regarding the biomass concentration, it has been considered as directly dependent on the photosynthetic rate. Similarly inorganic carbon concentration can be calculated by a mass balance to the liquid phase. In the gas phase a mass balance is established for the oxygen and for the carbon dioxide. For readers interested in these aspects, a complete description of the model and assumptions about these balances are given in [16].

The main variables involved in the process of photosynthesis of microalgae cultures—dissolved oxygen, pH of the culture, biomass concentration, temperature, concentration of total inorganic carbon, oxygen mole fraction, mole fraction of carbon dioxide, and CO_2_ losses—have been included in the model. To include the influence of dissolved oxygen concentration on the photosynthesis rate, an inhibition model has also been used. A schematic representation of the model is shown in Appendix A.

As the mathematical model used in the laboratory is based on fundamental principles instead of empirical equations [16], it can be applied to other PBR types. As a result, a complete dynamic simulation model is obtained, thus enhancing its applicability in the design and operation of microalgae-based processes.

## 4. Environmental Issues

### 4.1. Solar Radiation

Based on the equation of the Sun's position in the sky throughout the year, the amount of solar radiation on a surface at a particular tilt angle can be calculated theoretically as a function of latitude and day of the year. The virtual laboratory incorporates the simulation of the horizontal solar radiation (direct, diffuse, and global), that is the radiation measured on a horizontal surface. The solar radiation is considered by assuming the PBR is south-oriented to obtain the maximum solar insolation. It must be noted that no real data are used to simulate solar radiation, only synthetic values derived from a data model. Just by setting the latitude and the longitude of the PBR location and the clearness index it is possible to simulate solar radiation in a correct way. The clearness index represents the proportion of the extraterrestrial solar radiation that is lost due to absorption in the atmosphere. Broadly, this index is equal to the global solar radiation on the surface of the Earth divided by the extraterrestrial radiation at the top of the atmosphere. It varies from around 0.8 under the clearest conditions to near zero under overcast conditions. This index is useful to determine the local weather effects on the simulations or to define possible experiences in an easy way. We can, for instance, simulate and compare a set of cloudy or sunny days just by interactively changing the value of this index. It must be mention that the value of the clearness factor is fixed for each simulation. However the user can modify on-the-fly the value of this parameter using the corresponding editing field in the tool. Detailed information about the implemented solar radiation equations is available in [17].

### 4.2. Temperature Model

Temperature is a very important variable for the growth of microalgae, being difficult to control. In the virtual laboratory temperature variations can be simulated inside the reactor, so it is expected to be a useful tool for the simulation and design of microalgae PBRs.

Temperature profiles have been determined as a function of both operating (mass flow and inlet temperature) and environmental conditions (ambient temperature and solar radiation). Not only the mass flow and inlet temperature, but also the ambient temperature and solar radiation conditions have been taken into account.

The temperature mathematical model formulation includes heat conduction in the tube walls, convection inside the tube, and solar radiation. See [18] for details. This model combines theoretical concepts of thermodynamics with classical theoretical and empirical correlations of fluid mechanics and heat transfer.

The Volume Element Method (VEM) has been used to formulate the problem. In this way, the reactor pipes are divided into lumped volumes, such that only one time-dependent ordinary differential equation (ODE), based on the first law of thermodynamics, is produced for the temperature. Interactions between volumes are established through empirical heat transfer correlations for convection, conduction and radiation. Low computational demand with the inclusion of the main physical phenomena is the major advantage of the method used.

### 4.3. Control Architecture

PBRs are very complex systems with many inputs and outputs which allow a great range of operability to manage them. From a control point of view, in a PBR different variables such as the CO_2_ injection, the culture circulation rate or the culture temperature can be adjusted to maximize biomass production. Usually, PBR systems are controlled by means of on/off valves and thus classical on/off switching controllers are implemented. Also, system dynamics and disturbances are not considered in the control system design. In our lab, we try to improve the control architecture by including three low level PI control loops to manage the CO_2_ flow rate, the culture circulation rate, and the culture temperature. See Appendix A.

In the three controllers, the same methodology has been used [19]. First, models of the three actuators are identified (valves for the injection of CO_2_ and cold water, and a pump for the culture velocity), and then the controllers are designed. See [20] for details. In each loop, four possible control modes are available: manual, on/off, and PI with two modes (time-based and event-based). See [21] for details on the selected event-based strategy.

Regarding the harvesting, the tool allows the simulation of an ideal harvesting process using a manual control. The user can require a decrease in the biomass concentration at a particular time of the simulation.

## 5. The Photobioreactor Simulation Tool

This section describes the main features of the simulator. The simulation of a PBR system demands a graphical user interface (GUI) with a high degree of flexibility. The simulator has been programmed with EJS, a freeware open-source tool developed in Java, specifically designed for the creation of interactive dynamic simulations [22]. Users can access the laboratory by visiting the University Network of Interactive Labs [23] with standard Java-enabled web browsers.

### 5.1. Description of the Simulator

**Figure 4.**
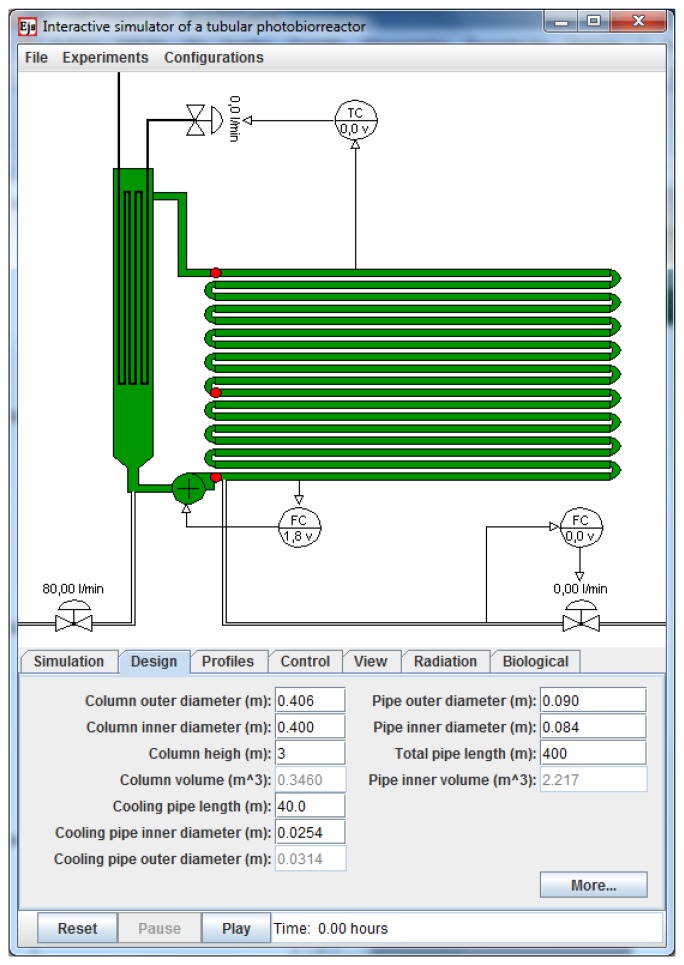
Graphical user interface of the PRB system.

Figure 4 shows the main window of the simulator. It displays the schematic representation of the PBR, which has been made as self-explanatory and realistic as possible. In the figure, the different components of the system can be clearly visualized. The mixing unit is represented by the vertically-oriented bubble column and the solar receiver is the horizontally-located tubular loop. In this simulation tool the fence-like vertically horizontal stacked PBR is simulated. It is not possible to simulate other configuration like for example a horizontal (on the ground laying) PBR. The color of the bubble column and the tubular loop varies from pale to dark green according to the instantaneous biomass concentration. Along the tubular loop, there are three red circles pointing to the places where the sensors of pH and temperature are located. Also, there is a centrifugal pump at the output of the bubble column to circulate the culture and pure CO_2_ is supplied on demand for pH control after the pump output. Before the centrifugal pump and at the bottom of the bubble column, there is a manual valve to supply air to the column (air flow rate) for the oxygen desorption.

**Figure 5.**
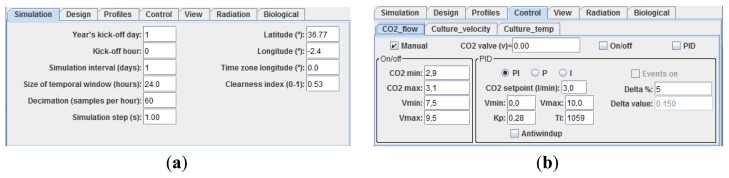
(**a**) *Simulation* Panel; (**b**) *Control* Panel.

At the bottom part of the main window there are six panels which allow not only changes in multiple parameters of the system but also the management of the system operation process in a proper way. In the *Simulation panel* different parameters associated with the global radiation model or the simulation interval in days can be modified interactively (see Figure 5a). When changes in physical parameters of the PBR are required such as the pipe and column lengths or the column and pipe diameters, *Design panel* must be selected (see lower part in Figure 4).

Three different control loops, represented in the main view, have been implemented in the simulator: (1) *loop to control the CO*_2_ *flow rate*, located below the tubular loop. This control is needed because the use of CO_2_ represents a major operational expense in microalgae culture which has to be minimized; (2) *loop to control the culture circulation rate* – the liquid circulation rate controls the turbulence in the system for light integration; it also determines the mixing behavior and the mass transfer capacity and it greatly avoids detrimental concentrations of dissolved oxygen in the tubes. It is located besides the centrifugal pump; and (3) *loop to control the culture temperature*, located at the top of the bubble column. In the *Control Panel* (see Figure 5b) the user can select the control operation mode making use of the three subpanels, one for each control loop. If the system is run in manual mode, the user needs to directly set, using the editable field provided to this end, the value of the manipulated variable. Obviously, the simulation addresses its main pedagogical target when it is set to a particular controller. The options available are manual, on/off, time-based PI, and SSOD PI control [21]. Depending on the selected controller the user can manage different parameters.

The simulator also provides in the *Profiles Panel* the possibility of loading sets of data obtained from previous real experiences. It allows users to conduct experiences using real information, not artificial values. For example, it is possible to load a file with real solar radiation data obtained from a weather database instead of using the artificial radiation data. It is also possible to select, using check boxes available in the *View Panel*, the time plot of the main system variables (biomass, pH, dissolved O_2_, global radiation, injected CO_2_, O_2_ productivity, PBR temperatures profiles or controller signals).

Other parameters related to the model system temperature or to the initial states of the main variables of the system (biomass concentration, pH, dissolved O_2_) can be also interactively changed in the *Radiation* and in the *Biological Panel*, respectively.

The components of the described interface provide the basic functionality required to operate the application. At the top of the main window, a menu bar provides some additional features. It allows users to load or save a simulation and to select a predefined experiment to be performed. The user can also save a configuration, saving the geometrical, operational, biological and radiation parameters of a particular simulation in independent files. The simulation can be played, paused, and reset using the standard push buttons of the interface.

### 5.2. A Practical Example

**Figure 6.**
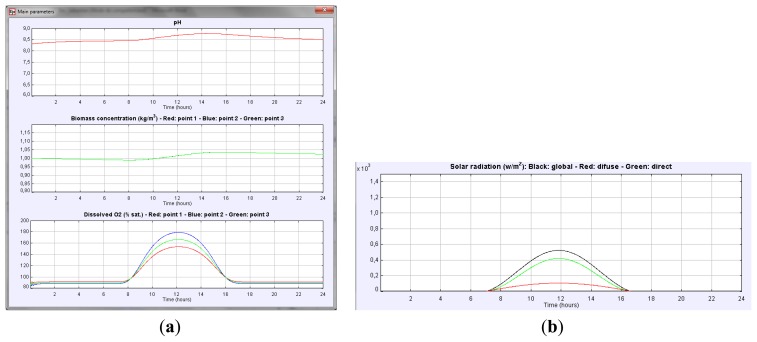
(**a**) Evolution of the main variables. (**b**) Intensity of the horizontal solar radiation in W/m^2^ on the PBR along the interval of days selected in the simulation.

This section presents a usage example of the simulator described above. Suppose that we want to measure the daily variation of the main variables such as culture pH, biomass concentration or dissolved oxygen in the culture for a particular PBR geometry, when no other parameters change. First the geometry is defined in the design panel, then the evolution of these variables can be observed just by selecting in the *View Panel* the corresponding variables (see Figure 6a).

As it can be observed, the dissolved O_2_ in the culture and the pH present a similar behavior. Dissolved O_2_ increases in the morning, reaching the maximum at midday, whereas in the afternoon, a decrease in dissolved O_2_ is observed due to a reduction of the photosynthesis rate. This variation indicates the existence of an oxygen accumulation term. Variation of culture pH is similar. The higher the solar irradiance is (see Figure 6b) the higher the pH of the culture is. Note that the solar radiation profile is easily obtained just by checking the global radiation button in the *View Panel*. The biomass concentration increases during the daylight period. Thus, accumulation of biomass in the reactor takes place.

**Figure 7.**
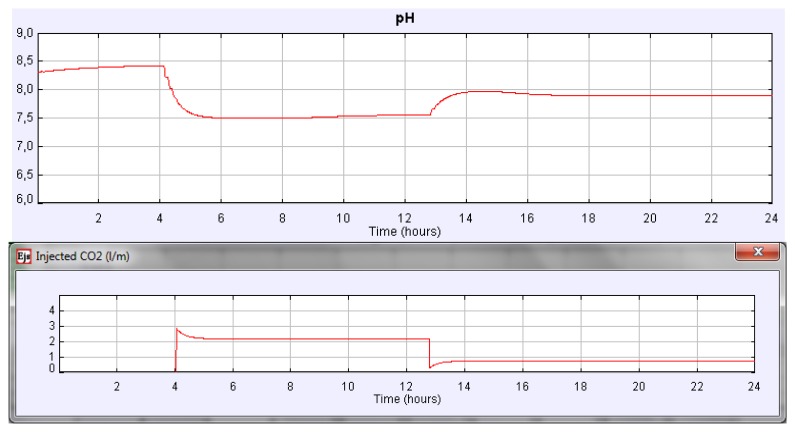
Effect in the culture pH when variation in the CO_2_ injected flow rate occurs.

The effect and control of the CO_2_ injected gas on daily variation of culture parameters can also be studied in an easy way. Figure 7 shows how the culture pH can be satisfactorily controlled with the increase and reduction CO_2_ flow rate in the injected gas. In this experience CO_2_ flow rate in the injected gas increased from 0 to 2.16 L/min at *t* = 4.15 h and then decreased from 2.16 to 0.72 L/min at *t* = 12.8 h using manual control. In this case, the steady state of the culture pH is 7.5 and 7.9 when the CO_2_ flow rate increases and decreases, respectively. However, if we address different experiences it can be noticed that the daily mean pH of the culture depends on the CO_2_ content. Nevertheless, similar daily variations in culture conditions are observed whatever the CO_2_ amount injected is.

**Figure 8.**
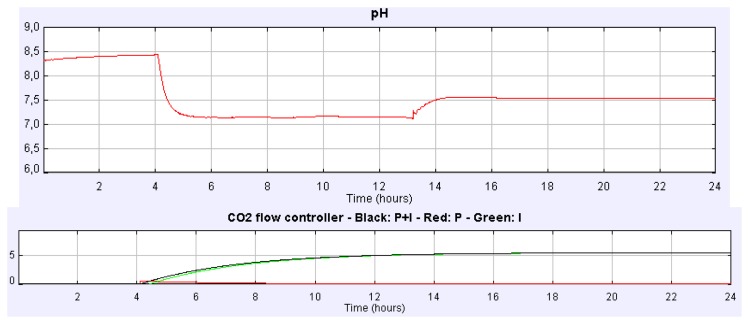
pH and controller signals evolution for the CO_2_ flow rate control using the PI time-based mode controller.

Suppose we address a similar experience setting the CO_2_ flow rate to 2.16 L/min but using instead the PI control implemented in time-based mode. Figure 8 displays the pH culture evolution for such a case. The steady state of the culture pH is reached again at 7.5. In Figure 8 the evolution of the controller signals can be observed.

**Figure 9.**
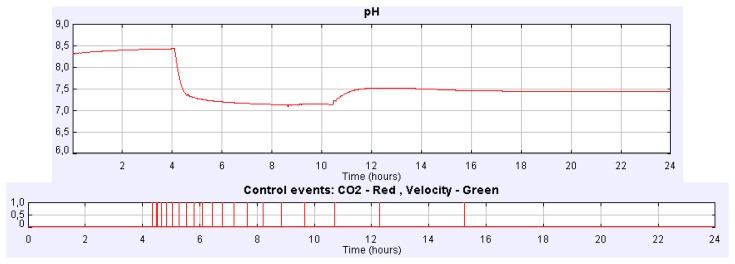
pH and controller signals evolution for the CO_2_ flow rate control using PI event-based mode.

If we carry out the same experiment but using the PI control implemented in event-based mode, the evolution of both pH and controller signals is the one showed in Figure 9. In this case the pH steady-state is reached at 7.5, but more than two hours before it is reached with the PI time-based controller. Many other experiments can be carried out with the simulator such as the influence of CO_2_ content of the injected gas on the analysis of the biomass or in the dissolved O_2_, the control of the culture temperature, the O_2_ productivity, *etc.*

## 6. Conclusions

PBRs are among the most promising culture systems for potential large-scale production of microalgae-derived high-value products. The clear necessity of developing innovative reactors to directly take into consideration the needs of the production of energy can be considered, for instance. Overall it can be said that many of the attempts to produce microalgae biomass have so far failed because of poor PBR engineering. There is a lack of scientific consensus on how a suitable and scalable PBR has to be designed [24]. In this sense, the optimal design and control of PBRs is an interesting and difficult control problem that is being widely analyzed.

This work focuses on the development of a virtual tubular PBR laboratory. To date, no other interactive simulator has been developed for PBR systems. The developed virtual lab is a valuable aid for both teaching and research in PBR systems. It helps to learn how a PBR works and to understand how the essential variables involved in the algae growth behave and interrelate between each other. As the culture productivity of a PBR is to a great extent dependent on both hydrodynamic and geometric parameters, using this interactive tool, the performance of a specific design can be analyzed. This can help either to address any issues that occur during algae growth or to provide solutions in the design of an optimal PBR, so using this tool it will be possible to simulate different controls over the growth environment resulting an interesting way of achieving the highest productivity.

Several works can be carried out to improve the laboratory in the near future. A more advanced harvesting process has to be developed to reproduce the real behavior of this operation. An automatic variation of the clearness index should also be included. New high level control strategies must be implemented to control different variables of the system. Using advances control strategies optimal culture conditions have to be determined in order to maximize biomass productivity. The implementation of Nonlinear Predictive Control has already been used in PBR systems to this end in previous works [25]. It would be nice to apply this methodology to our simulation tool. The level of nutrients is also an essential variable to be controlled in order to ensure optimized growth. Moreover, a tilted surface could be incorporated into the PBR system by modifying the solar radiation estimation properly, because now only horizontal solar radiation is taking into account in the model.

## Figures and Tables

**Figure A1.**
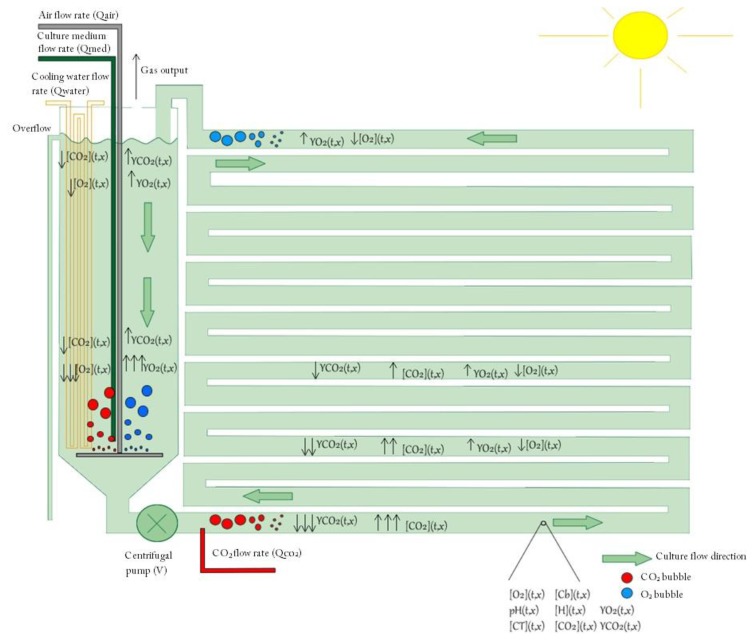
Schematic representation of the model.

## Appendix A

A schematic representation of the model is shown in Figure A1, where the nomenclature used is the following:

[CO_2_]: carbon dioxide concentration in the liquid phase
[O_2_]: oxygen concentration in the liquid phase
[Cb]: biomass concentration
CT: inorganic carbon concentration
YCO_2_: carbon dioxide to nitrogen molar ratio in the gas phase
YO_2_: oxygen molar ratio oxygen to nitrogen in the gas phase
PH : PH in the culture

A complete description of the growth model can be found in [16].

## Appendix B

In order to get a simulation as real as possible, models of the actuators used in the PBR located in the Almería facilities were included in the interactive simulator. First, to identify the operation points of each actuator many experiments were made by using the reaction curve method. So, incremental and decremental step inputs of the same amplitude were applied to each actuator. With all this information, the cooling water valve dynamics was approximated by first order systems with delay, the *CO*_2_ valve by a first order system with a zero, and the culture pump by dynamics of second order. After obtained the three transfer functions, these were validated with different data to the previously used in the identification phase in order to check that the simulated dynamics were similar to the real ones. In Table B1 the transfer function obtained for each actuation system is shown.

**Table B1.** Transfer functions of the identified models.

**Culture pump**	**CO_2_ valve**	**Cooling water valve**
$\frac{5.14}{\left(315.5s+1)(319.5s+1\right)}$	$\frac{3.6(1400s+1)}{1059s+1}$	$\frac{2.4}{81.9s+1}e^{-329s}$

As said before, the control of these actuators can be done by selecting manual or automatic operation. In case of choosing automatic, there are three options available to the user: on-off control, PI control with antiwindup mechanism, or event-based PI control (similar to PI controller but with a send of delta sampler located at the output of the sensor). Using the actuator models of Table B1 and the lambda tuning method, PI parameters for the three controllers were obtained. The transfer functions of the controllers are shown in Table B2.

**Table B2.** Transfer functions of the PI controllers.

**Culture pump**	**CO_2_ valve**	**Cooling water valve**
$0.39\left(1+\frac{1}{635s+1}\right)$	$0.28\left(1+\frac{1}{1059s+1}\right)$	$0.09\left(1+\frac{1}{81.9s+1}\right)$

Currently, the dynamics of the actuators presented in Table B1 cannot be changed by the user in the graphical user interface. In next versions, a new tabbed panel will be included to let users modify the dynamics depending of the actuators selected for designing their own PBR. Regarding the PI controllers, the user can only introduce new parameters values (*K_p_* and *T_i_*) but it will possible to included more elaborated controllers by adding to the simulator a tabbed panel to write their own control code.
